# Relaxation study of pre-densified silica glasses under 2.5 MeV electron irradiation

**DOI:** 10.1038/s41598-018-37751-9

**Published:** 2019-02-04

**Authors:** Nadège Ollier, Matthieu Lancry, Christine Martinet, Valérie Martinez, Sylvie Le Floch, Daniel Neuville

**Affiliations:** 1 Laboratoire des Solides Irradiés, CEA-CNRS Ecole Polytechnique, 91128 Palaiseau, France; 2 Institut de Chimie Moléculaire et des Matériaux d’Orsay, CNRS-Université Paris Sud, Université de Paris Saclay, Bât.410, 91405 Orsay, France; 3 Institut Lumière Matière, Univ Lyon, Université Claude Bernard Lyon 1, CNRS, F-69622 Villeurbanne, France; 4 Géomatériaux, CNRS-IPGP-USPC, 1 rue Jussieu, 75005 Paris, France

## Abstract

We examined the “relaxation properties” of pre-densified synthetic fused silica glass under 2.5 MeV electron irradiation. The densification of the glass was either obtained by hot compression (5 GPa-350 °C and 5 GPa-1000 °C) or via a thermal treatment increasing its fictive temperature (T_f_ = 1050, 1250 and 1400 °C). Under irradiation, the pre-densified silica glasses exhibit a relaxation of their macroscopic density with increasing integrated dose. Density was reduced for hot compressed silica and increased for T_f_ samples with different relaxation rates but it is remarkable that all sample densities follow a trend towards the same equilibrium value around 2.26 for a dose larger than 10 GGy despite a different final topology. After irradiation of hot compressed silica, the Raman spectra display a significant increment of 4 and almost 3-membered rings whereas they exhibit a glass density reduction; demonstrating that a D_2_ band increase cannot be considered as an absolute marker of the glass compaction. The correlation between density and D_2_ intensity remains valid until silica density remains lower than 2.26. In contrast, the FWHM of the main band peaking at 440 cm^−1^ appears to remain correlated to the silica glass density for all investigated samples.

## Introduction

Amorphous SiO_2_ is a glass model and remains among the most studied glasses due to its wide range of applications between Material and Earth Sciences. We can mention since 50 years the wide fields in optics and telecommunication while a huge work has been published on SiO_2_ glass under High-Pressure in links with geosciences. A high permanent densification on SiO_2_ glass can be reached after a High-Pressure treatment, for example, at room temperature from 25 GPa a densification rate of 21% has been obtained^1,2^. Another way to densify the glass permanently at lower pressure is to perform a hot compression, a review was recently published by Kapoor *et al*.^3^. Silica glass compaction can also be reached under different types of radiations (ion, neutrons, electrons)^4,5^ but with a lower efficiency compared to High Pressure. Despite the different nature of interaction mechanisms for neutrons and heavy ions (knock-on with atomic displacement) compared to electrons (bond breaking), the maximum of silica density variation does not exceed 3–4%^4,5^. The density was estimated to obey a power law as a function of the integrated dose D (α.D^β^ with β = 1 for neutrons and 2/3 for ionizing irradiation)^6,7^. UV (cw or ns)^8^ or fs laser^9^ is also well known to densify the silica glass with a compaction rate also lower than 3%. A two-photons mechanism (193 nm UV laser) was reported for Fiber Bragg gratings photo-inscription in optical fibers^10,11^.

The links between the glass density and microscopic glass structure are however not clear (especially in the reversible range lower than 10 GPa). The only accepted universal structural features describing the densification of silica is the reduction of the main Si-O-Si intertetrahedral angle but there is still existing controversy about the Si coordination number, Si-O bond length and rings size distribution despite the high number of experimental and calculations works on the subject. Recently, up to 13.5 GPa at ambient temperature, Trease *et al*.^12^ did not observe from their ^17^O NMR analyses any strong changes in Si coordination or ring size distribution. Concerning the ring size statistic, a few examples displayed opposite trends for D_2_ band area in silica glasses obtained after cold or hot compression but having the same macroscopic density^13^. Some recent papers have evidenced results ruling out the “classical image” of the destruction of high-membered size rings at the expenses of the low-membered rings (3 and 4). Huang and Kieffer^14^ showed an increase in number of larger rings at the expense of smaller rings by Molecular Dynamics simulation. Similarly, in Ge-doped silica samples having different fictive temperatures, the variation of density showed an opposite trend compared to pure silica in terms of D_1_ and D_2_ band intensity^15^. The D_1_ and D_2_ variation in case of lower density increment (<3%, typically those radiation-induced) is less debated. The association of silica glass compaction to an increase of D_1_ and almost D_2_ defect band intensity was reported for 2.5 MeV electrons^16^ or keV electrons^17^ where a linear increase of D_2_ area as a function of dose was shown. Under fs laser irradiation, the glass densification, often deduced from the silica refractive index increase is associated to the increment of the D_1_ and D_2_ Raman bands^18,19^. Recently, bond-breaking mechanism by IR fs laser interaction was modeled accurately by Molecular Dynamics by Shchleblanov and Povarnitsyn^20^. The increase of 3 and 4 membered rings with a reduction of 6 and 7 fold rings fraction was proposed to explain the densification of vitreous silica under femtosecond laser irradiation^20^.

This paper focuses on what we called “the relaxation properties” (density and structure probed by vibrational spectroscopy) of pre-densified silica glasses under electron irradiation while a previous one was dedicated to the point defects created in hot compressed silica glasses irradiated with 2.5 MeV glasses^21^. The present study is extended here by incorporating amorphous silica glass samples slightly densified (less than 1%) by increasing their initial fictive temperature (T_f_ = 1050, 1250 and 1400 °C) before subsequent irradiation. The main question addressed here is the impact of 2.5 MeV electrons ionizing irradiation (at multi GGy dose) on these densified silica glass structure relative to its density.

## Results

Table 1 reports the density values of the different Suprasil samples obtained either after a 5 GPa hot compression at 350 °C and 1000 °C or a T_f_ treatment (T_f_ = 1050, 1250 and 1400 °C). The evolution of the density values after 2.5 MeV electron irradiation can be read according to the integrated dose as well in this table.

**Table 1:** Density values of the different Suprasil samples obtained either after a 5 GPa hot compression at 350 °C and 1000 °C or a T_f_ treatment (T_f_ = 1050, 1250 and 1400 °C) and their evolution under irradiation for different integrated doses.

	Non-Irradiated (average value)	6.4 × 10^8^ Gy	2.4 × 10^9^ Gy	4.9 × 10^9^ Gy
Tf = 1050 °C	2.1996	2.205	2.208	2.219
Tf = 1250 °C	2.206	2.217	2.232	2.236
Tf = 1400 °C	2.212	2.222	2.242	2.248
5 GPa/1000 °C	2.612	2.580	2.484	2.317
5 GPa/350 °C	2.422	2.439	2.294	2.258

**Figure 1:**
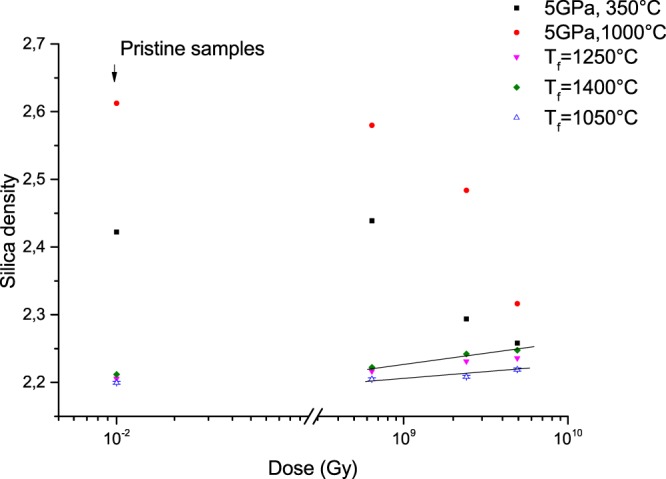
Evolution of the silica density for different pre-densified samples with the integrated dose (Gy).

In Fig. 1, the macroscopic silica glass density *ρ* is plotted as a function of the dose (log scale). The density value of the “pristine” samples is also reported, note that the silica glass density is 2.20 g/cm^3^. At first, we can observe two opposite trends with increasing dose: a reduction of density in case of HP-HT treated samples and a linear increase of density for all T_f_-treated silica samples. It is worth noticing that the slope (*Δρ*) for samples having a high fictive temperature T_f_ = 1400 °C is higher than those with T_f_ = 1050 °C. Moreover, both relaxation curves (5 GPa, 350 °C and T_f_ = 1400 °C) under irradiation exhibit a trend towards a similar density value around 2.25–2.26 (for the dose 4.9 × 10^9^ Gy). We assume that an equilibrium value (corresponding to a macroscopic density around 2.26) should be reached for all glasses at doses higher than 10 GGy.

**Figure 2:**
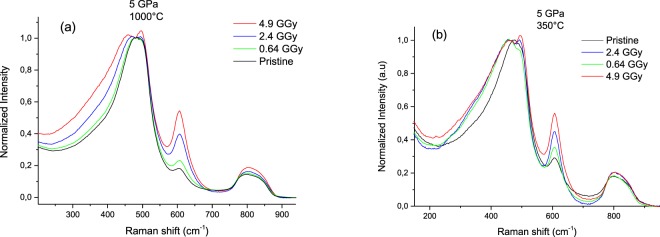
Raman spectra evolution with the integrated dose of (5 GPa, 1000 °C) (**a**) and (5 GPa, 350 °C) (**b**) samples.

Figure 2 displays the evolution with the integrated dose of the Raman spectra of two pre-densified silica glasses by HP-HT. They are labeled A and B respectively for (5 GPa, 1000 °C) and (5 GPa, 350 °C). The Raman spectra (normalized at the 440 cm^−1^ band intensity, also called R-band) show an enhancement of the FWHM of the 440 cm^−1^ band when the dose increases as well as a shift towards lower frequency. Both evolutions indicate a larger distribution of the average Si-O-Si angle as well as an increase of the average Si-O-Si angle value and are consistent with a decrease of the silica glass density. One can notice however that in case of (5 GPa, 1000 °C) the band broadens monotonically with the dose whereas the broadening is independent of the dose (0.64–4.9 GGy) for (5 GPa, 350 °C). Moreover, with increasing dose, the D_1_ and D_2_ defect band intensity peaking respectively at 495 and 606 cm^−1^ enhances monotonically in case of (5 GPa, 350 °C) sample. These bands correspond respectively to the in phase-O bending motion in fourfold and threefold rings, known as “breathing mode”^22^. The compressed glass at (5 GPa, 1000 °C) displays a similar behavior concerning the D_2_ band while the fourfold rings signature (D_1_ band) is enhanced only for the highest dose of 4.9 Gy.

**Figure 3:**
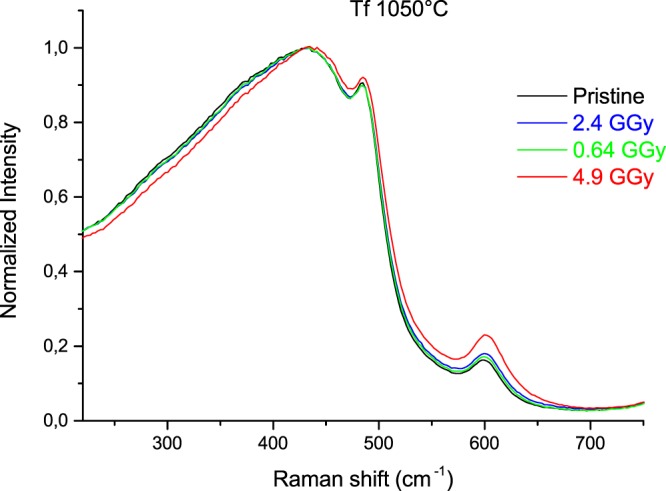
Raman spectra evolution with the integrated dose of (T_f_ = 1050 °C) samples.

**Figure 4:**
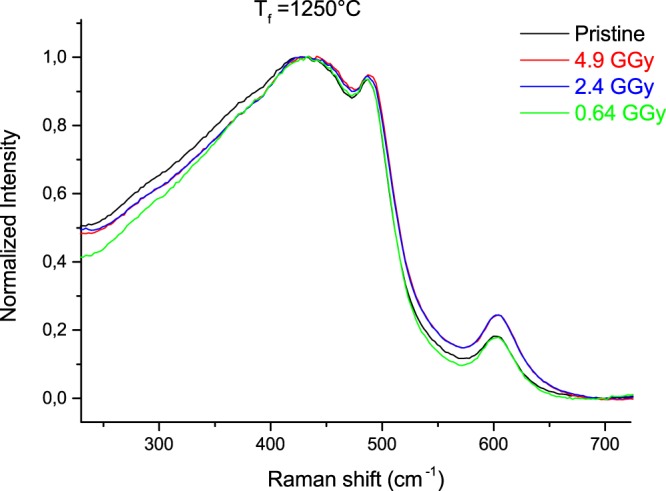
Raman spectra evolution with the integrated dose of (T_f_ = 1250 °C) samples.

**Figure 5:**
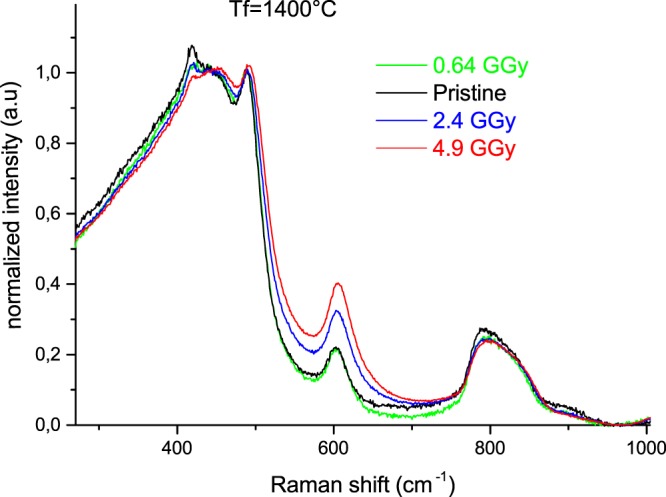
Raman spectra evolution with the integrated dose of (T_f_ = 1400 °C) samples.

The Raman spectra of thermally treated samples with corresponding T_f_ = 1050 °C, 1250 °C and 1400 °C and their evolution with integrated doses can be observed respectively in Figs 3, 4 and 5. For all fictive temperature treated samples, the main modifications with increasing dose are: a slight shift towards higher frequency as well as a small narrowing of the R band. The D_2_ band intensity is enhanced after 2.4 and 4.9 GGy doses irradiation. We can note a slight change in the pristine T_f_ = 1400 °C spectra (418 cm^−1^) attributed to the formation of a few nanocrystallites that are subsequently amorphized during the irradiation.

**Figure 6:**
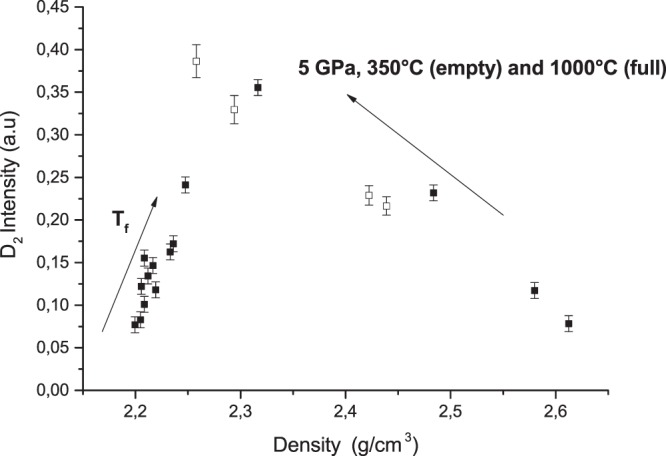
Raman D_2_ band intensity as a function of density for HP-HT and T_f_ samples.

**Figure 7:**
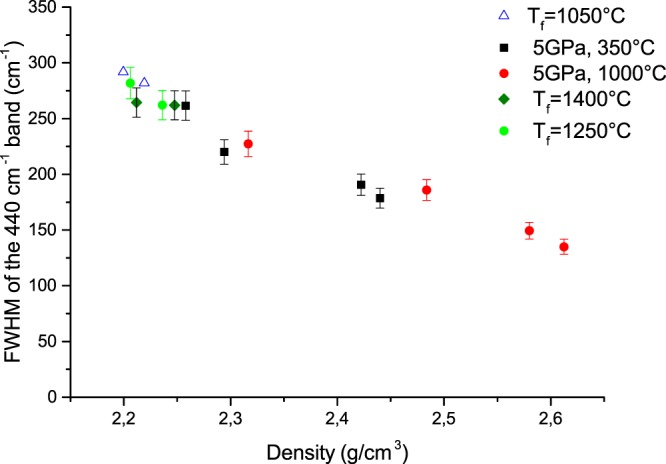
FWHM of the R-band as a function of density for HP-HT and T_f_ samples.

In order to better quantify the observed variations of all densified glasses (T_f_ and HP-HT) under irradiation, the FWHM of the 440 cm^−1^ band and the D_2_ intensity as a function of density are plotted in Figs 6 and 7 respectively. We can notice in Fig. 6 that for density values lower than 2.3, the D_2_ band intensity increases linearly with the density in all irradiated samples. However for higher densities (mostly related to irradiated HP-HT samples), the number of threefold rings appears to be anti-correlated to the glass density i.e. we observed an increase of D_2_ band whereas the glass density is decreasing. It demonstrates that two glasses with different macroscopic densities can exhibit a similar D_2_ band intensity. It infers that the enhancement of D_2_ band that is correlated to a larger number of small rings in the glass structure cannot be systematically associated to a silica glass densification. It remains true when the density does not exceed 2.26, the “equilibrium value” described in Fig. 1. The FWHM of the main band at 440 cm^−1^ versus the glass density is plotted in Fig. 7 for all irradiated samples. It can be seen that the FWHM i.e the Si-O-Si angle dispersion decreases when the silica glass density increases.

## Discussion

**Figure 8:**
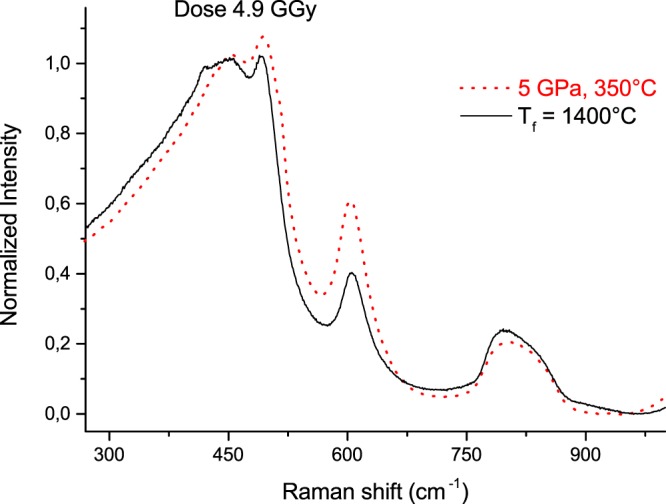
Raman spectra of (5 GPa, 350 °C) and T_f_ = 1400 °C both irradiated at 4.9 GGy displaying a close density value.

In this paper, we were interested in analyzing the relaxation effects under high-energy electron irradiation on pre-densified silica glasses obtained either from High Pressure-High Temperature (HP-HT) or from thermal treatment increasing their initial fictive temperature T_f_. We studied both the variation of macroscopic density as a function of the integrated dose as well as the glass structure by Raman spectroscopy. The compaction effect is enhanced with integrated dose in slightly densified glasses (i.e. with higher T_f_). In contrast for HP-HT samples, which own a higher initial density value (>2.40), the silica glass density is reduced by bonds breaking mechanisms. It is worth underlining that all “relaxation curves” converge towards the same “equilibrium” density value i.e. around 2.26. It is striking to note that this value corresponds to the density of amorphized quartz and amorphous silica irradiated by fast neutrons for integrated dose higher than 9 × 10^19^ n.cm^−2^ ^4,23^. Irrespective of the nature of the irradiation process, it was also reported for electron and ion irradiations that alpha-quartz converts progressively to a phase with the same density (about +3% than the common amorphous silica)^24^. Simulation studies (MD) by Anoop Krishnan *et al*.^25^ reproduces quite well the experiments on evolution of alpha-quartz density under 10^18^ neutrons except the final value that is questionable around 2.20 against 2.26 (the experimental one^4,23^). It is thus remarkable that the density of amorphous silica whatever its initial topology and density evolves under irradiation towards the same density, which is by the way the one of the α-tridymite silica polymorph^26^. Moreover, the nature of induced mechanisms e.g. knock-on (fast neutrons) or bond-breaking and point defects creation (electrons) is known to impact the kinetics of the silica densification with dose (proportional to D^β^ with β = 1 for neutrons and β = 0,6 for ionizing irradiation^5^). Note these differences in the β values reveal the different densification mechanisms. For a process occurring through short-range interactions at randomly distributed traps in a configuration space with a restricted dimensionality *d*, the parameter β is related to the dimensionality d^27^ following $\beta =\frac{d}{d+2}$. So, when *d* = 3, β is around 0.6 as for ionizing radiation indicating a kind of defects-assisted densification. In contrast when d → ∞: β → 1 as it is observed for the densification under neutrons; revealing in such a way that the process is not sensitive to any specific and pre-existing trapping sites. But it is remarkable to note that both kinds of irradiations follow a similar trend towards the same equilibrium density value. The final products however do not display equivalent vibrational structure between them or compared to the α-tridymite Orthorhombic form that is formed of 6 membered rings. Figure 8 illustrates this result in terms of intertetrahedral Si-O-Si average angle and rings statistic where (5 GPa, 350 °C) and T_f_ = 1400 °C treated samples, both irradiated at 4.9 GGy are compared. It means that the Si-O-Si average angle that can be extracted from the R band position can not be directly associated to the density, in agreement with previous results^5,13^. A similar Si-O-Si angle decrease is observed after HP or neutron irradiation (about −10° when irreversible structural change occurs) whereas very different densification efficiency are reached^5^. In ref.^13^, Martinet *et al*. noticed that hot and cold compressed silica having the same density display significantly different Raman spectra. This was also supported by recent thermal relaxation experiments on hot compressed silica samples showing non-equivalence between the structural microscopic evolution and the density relaxation^28^.

If we focus now on the D_2_ band variation in our pre-densified and subsequently irradiated samples, we observe first a positive correlation between the D_2_ band intensity and density when it is lower than 2.26. This is consistent with Gavenda *et al*.^17^, which reports a linear increase of the D_2_ band area with the dose (for compaction rate <3% and 15 keV electrons irradiation). Most of laser (IR fs or UV ns) related silica studies also (where density variation is less than 1%)^9,18,19^ mention a D_2_ band increase that is assigned to a glass densification. In contrast, for higher value than 2.26, which corresponds to the “equilibrium relaxed value”, the D_2_ increase with a decreasing density appears more counter-intuitive. However as reminded in introduction, this behavior is consistent with experimental and calculations studies on densified silica by HP^13,14^. Indeed for two densified silica glasses exhibiting the same density, D_2_ area depends strongly on the densification path (P, T)^13^. The population of 3-membered rings is increasing but the evolution of the R-band (asymmetric widening) shows that the populations of 6 and 7 membered rings increase also. This is in agreement with calculations of Trachenko and Dove^29^ showing a widening of the ring distribution and appearance of both larger and smaller rings. At the same time, the center of the rings distribution is shifting to larger rings so these dominate within the distribution. Furthermore, Guerette *et al*.^30^ who cross the results from XRD, Molecular Dynamics and Raman question the quantitative estimation of threefold rings from the D_2_ intensity. They mentioned that a D_2_ band increase intensity does not necessary reflect an increase of 3-membered rings population but a network rearrangement to which these rings are bonded and in which they vibrate^30^. Note that these measurements confirm that D_2_ increase cannot be considered as an absolute marker of glass densification whereas many scientists from the glass-irradiation community commonly use it. It remains only true in SiO_2_ if the density is below the saturation around 2.26. The FWHM of the R-band however seems to be a more relevant marker than D_2_ in agreement with the variation observed in ref.^13^.

Considering the relaxation rate under irradiation, we showed that with the applied dose it varies from one sample to another. The relaxation time for (5 GPa, 1000 °C) sample appears to be slower compared to (5 GPa, 350 °C). We suggest that due to 1000 °C temperature reached during the high pressure compression, the silica glass structure is likely exhibiting less disorder (i.e. a less wide Si-O-Si angles distribution as supported by the smaller FWHM of the R-band), which leads to a slower relaxation under electrons irradiation. This result is in agreement with the average relaxation time of Suprasil silica glasses compressed with a Belt Press with following conditions: 5 GPa, T = 1000 °C and 440 °C measured in ref.^28^. They estimated at 850 °C an average relaxation time of 9300 s^−1^ for (5 GPa, 1000 °C) against 410 s^−1^ for (5 GPa, 440 °C). This difference in terms of density relaxation rate is moreover consistent with the structural evolution of both glasses followed by Raman spectroscopy. We reported a difference in term of Si-O-Si angle dispersion (FWHM of the 440 cm^−1^ band) increase with dose. It appears that the most strained HP-HT sample (5 GPa, 350 °C) relax much faster leading to a similar FWHM whatever the integrated dose within 0.64–4.9 GGy range.

Our measurements also demonstrate that the sample originated at T_f_ = 1400 °C has a higher compaction rate under irradiation compared to the T_f_ = 1050 °C sample. This result is supported by the ionization-induced compaction model developed by Piao *et al*.^6,8^. The vitreous silica structure is frozen into a state characteristic of its fictive temperature with an excess of energy stored in the strained Si-O-Si bonds. This excess energy can act as a driving force for the relaxation process induced by breaking bonds explaining why the compaction rate for T_f_ = 1400 °C is higher than a lower initial fictive temperature namely T_f_ = 1050 °C.

**Figure 9:**
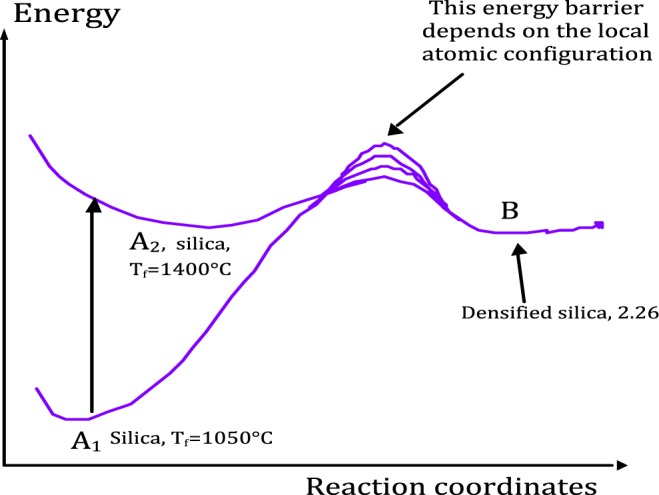
Chemical pathway between a pre-densified silica A and the final B state. Example of the influence of initial fictive temperature T_f_.

Poumellec has reviewed and presented an approach^31^ to account for such kinetics in glasses under irradiation or thermal treatment. It is shown that physical quantity (like the density) changes under irradiation (laser or others irradiations) can be explained by physico-chemical reactions dependent on disorder activation energy in glasses. As shown in Fig. 9, we can write the reaction A → B where A is related to the raw manufactured silica with a 2.20 density and B is the silica glass with a 2.26 density. We consider that the constant rate k(E) of this reaction is thermally activated and distributed. As a matter of fact, in glasses, *E* is not unique. The creation energy of “B silica state” can vary with its atomic environment. The transition state energy (the bottleneck on the chemical reaction pathway) is also sensitive to the disordered environment in such a way that the activation energy (energy difference between B and the transition state) is distributed with a distribution g_B_(E). This means that chemical pathways are variable depending on the configuration distribution of the transition state and/or the initial stable states throughout the glass i.e. initial T_f_ or HP-HT history. Note the densification reaction is thus faster in some places than in others leading to microscopic heterogeneities. Since the pre-densification process is not “saturated” (we did not reach yet the equilibrium value of 2.26 using the T_f_-treatment), which would lead to the final B state, its distribution function g_B_(E) is not saturated and, thus, neither is the subsequent irradiation-induced densification reaction. The densification rate under irradiation is thus dependent on the pre-densification (i.e. time and temperature for T_f_ samples but also for time, T and P for HP-HT samples). This should lead to a higher compaction rate for a high T_f_ sample. According to this approach, any variation of the initial distribution (prior irradiations) modifies the relaxation kinetics under irradiation. In this framework we can also explain the so-called relaxation under irradiation, which is faster for a (5 GPa, 350 °C) sample than for (5 GPa, 1000 °C) HP-HT sample. Indeed the initial distributions (prior irradiation) are obviously different for these two samples, which is confirmed by the respective thermal relaxation measurements performed at different temperatures in ref.^28^.

Except the relaxation studies under irradiation of highly densified silica phase i.e. α-quartz^23,24,32^ a few examples of thermal relaxation of densified silica can be found in literature as already discussed above. Gavenda *et al*.^17^ investigate the thermal relaxation of the D_2_ band area between 600 and 900 °C in electron-irradiated silica samples and they reveal a complete recovery up to the level of pristine silica glass. Cornet *et al*.^28^ analyzed the thermal relaxation of both hot compressed and cold compressed silica samples. They evidenced an important D_2_ increase during an initial step of the relaxation process while the density monotonically decreases down to its initial value around 2.20. This is attributed to a physico-chemical pathway going through a “transitory state” associated to an increase of the network inhomogeneity. However no transitory state was evidenced in our experiments (i.e. relaxation under electron irradiation) where D_2_ follows a monotonic increase in both 350 °C and 1000 °C HP-HT samples with the integrated dose. But the final products reached at the end of the process are different: standard 2.20 density silica in the case of thermal relaxation^27^ vs. 2.26 density silica for the “relaxation-induced by electron irradiations”. However it is worth to note the similarity between both relaxations behaviors displaying an increase of threefold rings while the density is decreasing. This similarity could be inferred if the inflexion point of the D_2_ curve delimiting the two regimes in Fig. 5 from^28^ was associated to a 2.26 density value, which should be investigated in future experiments.

## Method

In this study we used high purity synthetic fused silica materials manufactured by flame hydrolysis of SiCl_4_. Heraeus Suprasil F300 silica rod samples (<1 ppm of OH, 2000 ppm Cl) were densified by applying High Pressure (5 GPa) and High Temperature (350 and 1000 °C) in a belt press, at the ILM Laboratoire in the PLECE Platform at Lyon 1 University, described into details in ref.^13^. The concept of fictive temperature was first introduced by Tool^33^, and defined it as “the temperature at which the glass would find itself in equilibrium if suddenly brought to it from its given state”. It describes thus the structure of a glass and is related to the cooling rate or more generally a thermal history^34^, which can be changed (from its “raw manufactured” value) with a dedicated annealing. Careful temperature annealing treatments and subsequent quenching as described in^34^ were carried out on Heraeus Suprasil 1 samples (1000–1200 ppm OH, 100 ppm Cl) allowing fixing their fictive temperatures (T_f_ = 1050, 1200 and 1400 °C) and densify them compared to pristine silica.

2.5 MeV electron irradiation was then performed on SIRIUS electron accelerator facility at LSI (Laboratoire des Solides Irradiés), Palaiseau, France, supported by the National Network EMIR. 3 different doses (6 × 10^8^ Gy, 2.9 × 10^9^ Gy and 4.9 × 10^9^ Gy) have been integrated at 300 K on these samples. The thickness (typ. < 800 μm) allows a homogeneous dose deposit within the whole sample volume.

Densities of all samples were measured with the ‘sink-float method’ based on the Archimedian technique using toluene as an immersion liquid. The relation between these weights and density and temperature of toluene is well-known (density(T) = 0,8845–0,9159 × 10^−3^xT + 0,368.10^−6^ xT^2^, T in °C)^35^. For each sample, density measurements were performed 3 times before and after irradiations, resulting in an error of approximately 10^−3^.

All Raman spectra were performed at room temperature in MONARIS Lab (Paris 6) using a Labram HR micro-spectrometer (from Horiba Jobin-Yvon) equipped with a 458 nm Argon laser (6 mW on the sample) and a x100 microscope objective from Olympus.
